# Serum advanced glycation end products as a putative biomarker in Type2 DKD patients’ prognosis

**DOI:** 10.3389/fphys.2025.1541198

**Published:** 2025-01-31

**Authors:** Ze-Hou Wang, Zong-Jin Zhang, Yue-Fen Wang, Jin Xie, Yi-Min Li, Cun Shen, Yuan Meng, Wen-Jing Zhao, Lu-Ying Sun, Wei Jing Liu

**Affiliations:** ^1^ Department of Nephrology, Dongzhimen Hospital Beijing University of Chinese Medicine, Beijing, China; ^2^ Beijing University of Chinese Medicine, Beijing, China; ^3^ Department of Traditional Chinese Medicine, Majiapu Community Health Service Center, Beijing, China; ^4^ Department of Nephrology, Beijing Hospital of Traditional Chinese Medicine, Capital Medical University, Beijing, China; ^5^ Department of Geriatrics, Nantong Traditional Chinese Medicine Hospital, Nantong, China; ^6^ Department of Nephrology, Fangshan Hospital Beijing University of Chinese Medicine, Beijing, China; ^7^ Key Laboratory of Chinese Internal Medicine of Ministry of Education and Beijing, Dongzhimen Hospital Affiliated to Beijing University of Chinese Medicine, Beijing University of Chinese Medicine, Beijing, China

**Keywords:** advanced glycation end products (AGEs), diabetic kidney disease (DKD), corrected lgAGEs, urinary albumin-to-creatinine ratio (UACR), renal prognosis

## Abstract

**Aim:**

Advanced glycation end products (AGEs) are pivotal mediators in diabetic kidney disease (DKD). However, their prognostic utility remains underexplored. This study introduced corrected lgAGEs [novel biomarker derived by adjusting logarithmically transformed AGEs (lgAGEs) levels based on serum albumin (ALB) levels] to enhance the prediction of adverse renal outcomes in patients with type 2 DKD (T2DKD).

**Methods:**

In this prospective cohort study, 196 T2DKD patients were followed up longitudinally. Serum AGEs levels were log-transformed and adjusted for ALB to calculate corrected lgAGEs. Participants were stratified into the high- and low-level groups based on the median corrected lgAGEs. The association between corrected lgAGEs and renal outcomes was assessed using Cox proportional hazards models. Receiver operating characteristic (ROC) curve was utilized to evaluate the predictive performance of corrected lgAGEs alone and in combination with the urinary albumin-to-creatinine ratio (UACR).

**Results:**

High level of corrected lgAGEs was independently associated with adverse renal outcomes [hazard ratio (HR), 3.252; 95% confidence interval (CI), 1.461–7.243; *p* = 0.003]. Kaplan-Meier analysis demonstrated that patients in the high-level group (12 months) exhibited significantly shorter median survival times compared with those in the low-level group (50 months). ROC analysis showed that UACR alone had an area under the curve (AUC) of 0.782 (95% CI, 0.705–0.858), with 82.8% sensitivity and 61.5% specificity. Corrected lgAGEs achieved an AUC of 0.725 (95% CI, 0.637–0.814), with 69.0% sensitivity and 76.9% specificity. Combining UACR and corrected lgAGEs improved the specificity to 75.6%, with an AUC of 0.764 (95% CI, 0.682–0.847), while maintaining a sensitivity of 70.7%.

**Conclusion:**

Corrected lgAGEs are novel and independent biomarkers for predicting adverse renal outcomes in T2DKD. Combining UACR with corrected lgAGEs could enhance risk stratification by improving the specificity, highlighting its potential application in early identification of high-risk patients. These findings should be validated in broader populations in future research.

## 1 Introduction

Diabetic kidney disease (DKD) is one of the most common microvascular complications of diabetes. According to the International Diabetes Federation, China has the largest population of diabetes, with an estimated 140 million in 2021 and an incidence rate of DKD of 20%–40%; the number of people with diabetes will exceed 174 million by 2045 (Sun et al., 2022). DKD has a high incidence and disability rate, resulting in significant medical and societal costs. DKD is primarily characterized by chronic proteinuria and a steady reduction in glomerular filtration rate (GFR) (Yamazaki et al., 2021), which can progress to end-stage renal disease (ESRD). Urinary albumin-to-creatinine ratio (UACR) and estimated glomerular filtration rate (eGFR) are common indicators for diagnosing and evaluating the severity of DKD (Jung and Yoo, 2022). However, these indicators are often influenced by various variables and frequently fail to adequately reflect the prognosis. Therefore, it is of great value to identify innovative, more sensitive, and specific biomarkers that can indicate the prognosis of DKD patients.

Metabolic memory plays a significant role in the pathophysiology of microvascular problems in diabetes. Advanced glycosylation end products (AGEs) can bind to the receptor for advanced glycation end products (RAGE), triggering the subsequent cascade reactions (Rabbani and Thornalley, 2021; Khalid et al., 2022). Chronic hyperglycemia can cause long-term renal damage, leading to DKD. AGEs and RAGE play important pathogenic roles in DKD pathogenesis and progression, activation of RAGE by AGEs triggers oxidative stress and inflammation through downstream signaling pathways, including nuclear factor-kappa B (NF-κB),Erk1/2 and p38 MAP kinases. This cascade leads to upregulation of pro-inflammatory cytokines, such as tumor necrosis factor-alpha (TNF-α) and interleukin-6 (IL-6), as well as increased expression of vascular endothelial growth factor (VEGF) and adhesion molecules (Rabbani and Thornalley, 2018). These molecular events collectively contribute to endothelial dysfunction, mesangial expansion, podocyte injury, and glomerulosclerosis. Moreover, dicarbonyl stress, characterized by elevated levels of reactive dicarbonyl compounds like methylglyoxal due to impaired glyoxalase-1 (Glo1) activity, exacerbates AGE formation and renal injury (Xue et al., 2012). Experimental models demonstrate that Glo1 overexpression mitigates DKD progression by reducing AGE accumulation and associated inflammation (Rabbani and Thornalley, 2018). Therapeutic strategies targeting the AGE-RAGE axis, including RAGE inhibition and enhancement of Glo1 activity, have shown promise in alleviating renal damage in preclinical studies. These findings underscore the central role of AGEs in DKD pathogenesis and highlight their potential as therapeutic targets.

In addition to their established role in disease pathogenesis, AGEs have been shown to outperform glycated hemoglobin (HbA1c) in predicting the onset of chronic diabetic complications, such as nephropathy and retinopathy (Monnier et al., 1999). Furthermore, AGEs have also been identified as potential biomarkers associated with cardiovascular risk, all-cause mortality, and progressive renal decline among individuals with diabetes (Koska et al., 2018; Sabbatinelli et al., 2022; Steenbeke et al., 2022). However, there is still scarce and inconclusive clinical evidence directly linking AGEs to kidney-specific outcomes in DKD, which is partly attributed to confounding factors such as the influence of exogenous AGEs (Busch et al., 2006).

It has been suggested that AGEs may show limited predictive value for disease prognosis when used alone, as they represent only one component of the broader AGEs-RAGE axis (Prasad et al., 2016). However, propose people proposing this hypothesis have not attempted to statistically evaluate AGEs alone in predicting disease outcomes. Nevertheless, emerging evidence has indicated that AGE levels are correlated with the severity of pathogenic alterations in DKD (Bronowicka-Szydełko et al., 2021; Koska et al., 2022), underscoring their potential as novel prognostic markers. AGEs are medium-molecular-weight toxin-protein complexes, which are known to accumulate in response to chronic hyperglycemia and oxidative stress. Importantly, there is currently no study clarifying whether the level of AGEs differs among individuals with varying serum ALB concentrations, a factor that could influence their clinical utility. Prior research has indicated that serum AGE levels in DKD patients are positively associated with eGFR, but these findings are limited by the exclusion of patients with significant proteinuria and hypoalbuminemia. This gap highlights the necessity for further investigation to validate the prognostic role of AGEs in different patient populations and to assess their utility in combination with other biomarkers for DKD.

In this prospective cohort study, we aimed to address the multifaceted role of AGEs in the prognosis of T2DKD. Based on the recognition of the potential impact of serum ALB on AGE levels, we introduced corrected lgAGEs, a novel measure calculated by adjusting logarithmically transformed AGEs (lgAGEs) for ALB concentration. Specifically, the objectives of this study were: (i) to identify independent determinants of serum lgAGEs; (ii) to examine the influence of eGFR and ALB stages on corrected lgAGEs; (iii) to evaluate whether elevated corrected lgAGEs levels can independently predict adverse renal outcomes in patients with T2DKD, and (iv) to assess the potential utility of corrected lgAGEs as a prognostic biomarker for disease progression. By allowing the inclusion of patients across a wide spectrum of serum creatinine (SCr), UACR, and ALB levels, we aimed to enhance the generalizability of our findings and provide new insights into the prognostic implications of AGEs in DKD.

## 2 Materials and methods

### 2.1 Study populations

This prospective study enrolled 196 patients with T2DKD (aged 18–80 years) who were recruited at the Beijing Hospital of Traditional Chinese Medicine from October 2016 to December 2021. Diagnosis and staging of DKD followed the 2024 Kidney Disease: Improving Global Outcomes (KDIGO) Guidelines (2024). T2DKD is diagnosed in patients with UACR ≥30 mg/g and/or eGFR ≤60 mL/min/1.73 m^2^ with a major disease of type 2 diabetes mellitus (DM).

Patients with type 1 diabetes, pregnancy, active infections, malignancies, or autoimmune diseases were excluded from the present study. Additionally, individuals with suspected secondary causes of kidney disease (such as urinary tract infections, polycystic kidney disease, and hematuria) or a history of glomerulonephritis were also excluded. Furthermore, participants with conditions that may influence serum AGE levels were also not eligible for inclusion, including those with indwelling catheters, active liver disease, or those receiving corticosteroids or immunosuppressive therapy.

**FIGURE 1:**
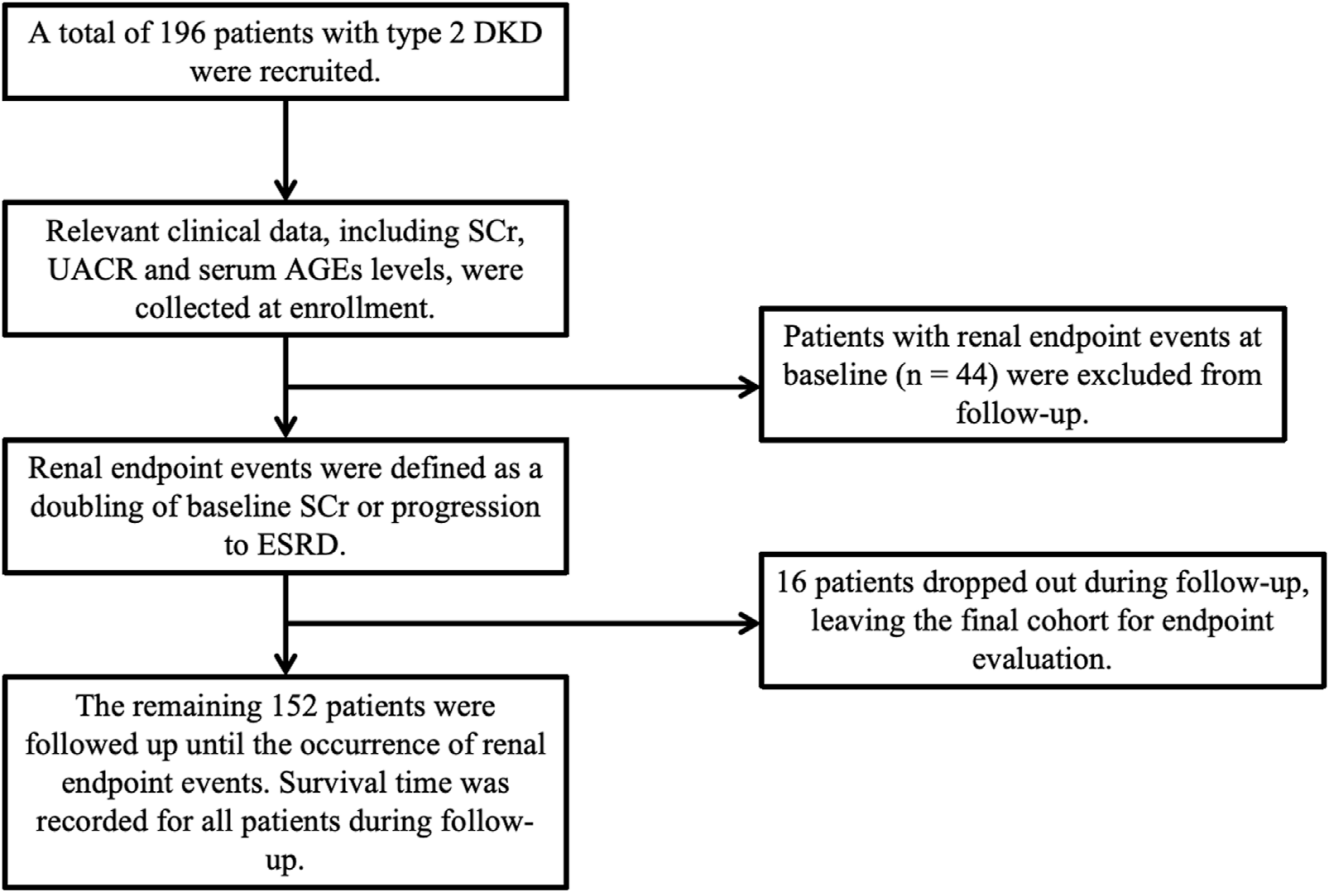
Trial flow.

Among the included 196 participants, 44 had developed ESRD at baseline, and the remaining 152 patients were enrolled in the cohort study, as depicted in Figure 1. Participants were followed up monthly during the first 3 months and then followed up every 6 months to 1 year, with their SCr and other relevant clinical data collected at each visit. Follow-up continued until the occurrence of a renal endpoint or the study’s conclusion. The primary renal endpoint was defined as a two-fold increase in baseline SCr or progression to ESRD. Survival time was calculated as the time from baseline to the occurrence of the renal endpoint.

The research plan was examined and approved by the Medical Ethics Committee of the Beijing Hospital of Traditional Chinese Medicine. All techniques were carried out in compliance with the standards and regulations of the Medical Ethics Committee of Beijing Hospital of Traditional Chinese Medicine.

### 2.2 Clinical data and detection index

Based on the clinical specimen bank of DKD patients in the Department of Nephropathy, Beijing Hospital of Traditional Chinese Medicine, the serum samples corresponding to the patients in the stages were obtained. Serum AGE levels were detected using an enzyme-linked immunosorbent assay (ELISA) kit (CEB353Ge, Wuhan Cloud Clone Company) their contents were recorded. To address the non-normal distribution of serum AGEs, we applied a log10 transformation followed by correction for serum ALB:

$$corrected\;lgAGEs=\log10\left(AGEs\right)÷albumin.$$

Logarithmic transformation is a well-established method to reduce skewness in data distribution, enabling more robust statistical analysis. Through applying this transformation, we preserved the relative relationships within the dataset. The subsequent correction for ALB was based on the role of ALB as the most abundant plasma protein and a primary target of glycation. ALB levels fluctuate significantly in pathological conditions such as proteinuria and hypoalbuminemia, which are common in DKD patients. Additionally, increased endothelial permeability in DKD can lead to protein redistribution, further affecting serum protein profile (Edriss et al., 2020). The method of normalizing AGE levels to ALB adjusts for such variability, ensuring that the measured AGEs more accurately reflect the glycation burden rather than protein concentration differences. This dual-step method enhances the reliability and interpretability of AGEs as biomarkers for DKD prognosis, providing advantages over traditional methods by addressing both distributional and protein-related issues.

### 2.3 Statistical analysis

Baseline characteristics were summarized as means ± standard deviation (SD) for normally distributed continuous variables, as medians with interquartile ranges for non-normally distributed continuous variables, and as frequencies with percentages for categorical variables. The distribution of corrected lgAGEs was assessed using the Shapiro-Wilk test, which indicated a non-normal distribution (*p* < 0.001). Consequently, non-parametric tests were employed to evaluate group differences. Specifically, comparisons between two groups were analyzed utilizing the Mann-Whitney *U* test, and those among multiple groups were analyzed using the Kruskal–Wallis test with *post hoc* Dunn–Bonferroni test to adjust for multiple comparisons, with individual *p* values reported. Based on baseline corrected lgAGEs levels, with the median value of 93.65 as the cut-off, participants were classified into the high-level (≥93.65) and low-level (<93.65) corrected lgAGEs stage groups. The survival rates between the two groups were compared using Kaplan-Meier survival analysis. Additionally, Cox proportional hazards regression was employed to estimate risk ratios, and multivariate Cox regression was applied to control for potential confounders. A two-sided *p*-value of <0.05 was considered a statistically significant difference. All statistical analyses were conducted using SPSS version 26.0 (IBM Corp., Armonk, NY).

### 2.4 Ethics approval and consent to participate

The research was conducted following the Declaration of Helsinki. The research scheme was reviewed and approved by the Medical Ethics Committee of Beijing Hospital of Traditional Chinese Medicine (reference number: 2017BL02-046-02). After fully explaining the purpose and nature of the research to the patients, all patients in the research signed an informed consent form.

## 3 Results

### 3.1 Baseline characteristics

After excluding patients with inadequate baseline medical records and no serum samples at baseline, a total of 196 individuals who matched the inclusion criteria were included. Among all the participants, 69.39% were male, with a median age of 61 years and a median diabetes duration of 192 months; the median eGFR was 35.50 mL/min/1.73 m^2^, while the median UACR was 2802.41 mg/g. The overall circumstances and baseline data of patients are shown in Table 1.

**TABLE 1:** Baseline characteristics of 196 T2DKD patients.

	High (n = 98)	Low (n = 98)	*p*
Age (years)	59.0 (12.2)	61.1 (12.5)	0.235
Gender (Male, %)	67 (68.4)	69 (70.4)	0.877
Diabetes course (months)	187.4 (89.5)	207.1 (97.7)	0.151
BMI(kg/m^2^)	26.0 (3.2)	24.9 (2.9)	0.146
Smoking (Yes,%)	40 (40.8)	42 (42.9)	0.885
Drinking (Yes, %)	32 (32.7)	25 (25.5)	0.345
Diabetic retinopathy (Yes, %)	76 (81.7)	62 (65.3)	0.017**
Diabetic peripheral neuropathy (Yes, %)	46 (49.5)	52 (56.5)	0.415
Diabetic foot (Yes, %)	7 (7.8)	6 (6.7)	1
Hypertension (Yes, %)	89 (91.8)	85 (89.7)	0.926
GFRstage (1/2/3/4/5, number)	5/10/31/24/28	21/16/30/15/16	0.002***
Albuminuria	97 (99.0)	68 (69.4)	<0.001***
AGEs (ng/mL)	1537.9 (755.7)	1462.5 (895.2)	0.525
HbA1c (%)	6.7 (1.4)	7.3 (1.5)	0.004***
Glu (mmol/L)	7.1 (2.9)	8.4 (3.4)	0.004***
SCr(µmol/L)	274.2 (198.4)	199.2 (189.9)	0.007***
eGFR (mL/min/1.73m^2^)	33.5 (24.9)	53.1 (35.3)	<0.001***
UA (µmol/L)	418.3 (90.7)	411.9 (85.5)	0.608
UREA (mmol/L)	14.0 (7.7)	30.7 (186.3)	0.378
CHO(mmol/L)	6.3 (3.5)	7.7 (30.6)	0.658
TG (mmol/L)	2.1 (1.7)	2.1 (1.8)	0.818
UACR (mg/g)	4689.6 (2629.6)	1746.9 (1858.2)	<0.001***
24 h-UTP (mg)	5943.0 (3973.7)	2077.3 (2236.1)	<0.001***
ALB (g/L)	26.8 (5.3)	39.0 (5.0)	<0.001***
Hgb(g/L)	106.2 (22.2)	120.7 (23.9)	<0.001***
PA (mg/L)	249.5 (68.6)	262.6 (66.1)	0.26

Values were expressed mean ± SD, n (%), or median (interquartile range) unless otherwise stated. Due to insufficient data for patients with stage A1 (only 7), the analysis of patients with stages A1 and A2 was combined to reduce potential errors. BMI, body mass index; Glu, abdominal blood glucose; HbAlc, glycosylated hemoglobin; UREA, urea nitrogen; SCr, serum creatinine; eGFR, was calculated according to CKD-EPI, formula and recorded in the database; UA, uric acid; CHO, cholesterol; TG, triglyceride; UACR, urinary albumin to creatinine ratio; 24 h-UTP, 24-h urinary protein quantification; ALB, albumin; PA, prealbumin.

### 3.2 Analysis of related factors of corrected lgAGEs

The correlations of lgAGEs were analyzed. As indicated by univariate correlation analysis results, corrected lgAGEs were negatively correlated with Glu, HbAlc, eGFR, and Hgb (correlation coefficients: 0.209, −0.199, −0.346, and −0.336, respectively) and positively correlated with diabetic retinopathy, UREA, SCr, cholesterol (CHO), UACR, and 24 h-urine total protein (UTP) (correlation coefficients: 0.187, 0264, 0.353, 0.377, 0.657, and 0.683, respectively) (all *p* < 0.05, Table 2). The components listed above were included in the multifactorial correlation analysis, which showed that UACR and 24 h-UTP had independent effects on corrected lgAGEs (Table 3).

**TABLE 2:** Correlation between corrected lgAGEs and baseline clinical related indicators.

Baseline clinical related indicators	Corrected lgAGEs (n = 196)	
	*r*s	*P* Value
Gender	0.002	0.974
Ages(years)	−0.062	0.39
Diabetes course (months)	−0.121	0.098
BMI(kg/m^2^)	0.088	0.469
Smoking	0.021	0.766
Drinking	0.061	0.397
Diabetic retinopathy	0.187	0.01**
Diabetic peripheral neuropathy	−0.082	0.266
Diabetic foot	0.008	0.913
Hypertension	0.077	0.286
HbA1c (%)	−0.199	0.006***
Glu (mmol/L)	−0.209	0.004***
SCr(µmol/L)	0.353	<0.001***
eGFR (mL/min/1.73m^2^)	−0.346	<0.001***
UA (µmol/L)	0.062	0.392
UREA (mmol/L)	0.264	<0.001***
CHO(mmol/L)	**0.377**	<0.001***
TG (mmol/L)	0.056	0.437
UACR (mg/g)	0.657	<0.001***
24 h-UTP (mg)	0.683	<0.001***
Hgb(g/L)	−0.336	<0.001***
PA (mg/L)	−0.147	0.088

Data were analyzed using the Spearman test.

Bivariate correlation indicated that Glu, HbAlc, eGFR, and Hgb had a negative correlation with corrected lgAGEs; diabetic retinopathy, UREA, SCr, CHO, UACR, and 24 h-UTP, had a positive correlation with corrected lgAGEs (p < 0.05).

**TABLE 3:** Multiple linear regression analysis of corrected lgAGEs.

Independent variable	Non-standardized β	S.E	Beta	t	*P* Value
Diabetic retinopathy	0.619	4.324	0.009	0.143	0.886
HbA1c (%)	0.753	1.499	0.038	0.502	0.616
Glu (mmol/L)	−0.405	0.665	−0.045	−0.609	0.543
Scr(µmol/L)	−0.018	0.013	−0.122	−1.352	0.178
eGFR (mL/min/1.73m^2^)	0.007	0.101	0.007	0.068	0.946
UREA (mmol/L)	−0.011	0.013	−0.051	−0.834	0.405
CHO(mmol/L)	−0.019	0.082	−0.014	−0.231	0.818
UACR (mg/g)	0.004	0.001	0.352	4.43	<0.001***
24 h-UTP (mg)	0.002	0.001	0.299	3.793	<0.001***
Hgb(g/L)	−0.193	0.099	−0.159	−1.95	0.053

Multiple linear regression was used to verify the positive and negative bivariate correlation results. Multiple linear regression indicated that the increases of UACR, and 24 h-UTP, had a positive effect on the content of corrected lgAGEs (p < 0.05).

Additionally, correlation analysis showed that AGEs were negatively correlated with SCr, UA, and UREA (correlation coefficients: 0.299, −0.253, and −0.187, respectively) and positively correlated with eGFR, Hgb, and Alb (correlation coefficients: 0.282, 0.177, and 0.208, respectively) (all *p* < 0.05). Furthermore, ALB had an independent influence on AGEs (Supplementary Material).

### 3.3 Comparison of corrected lgAGEs in different stages

Based on the GFR (G) and albuminuria (A) stages proposed in the 2012 KDIGO clinical practice guide for chronic kidney disease (CKD) evaluation and management, patients in this study were divided into different G and A stages.

**FIGURE 2:**
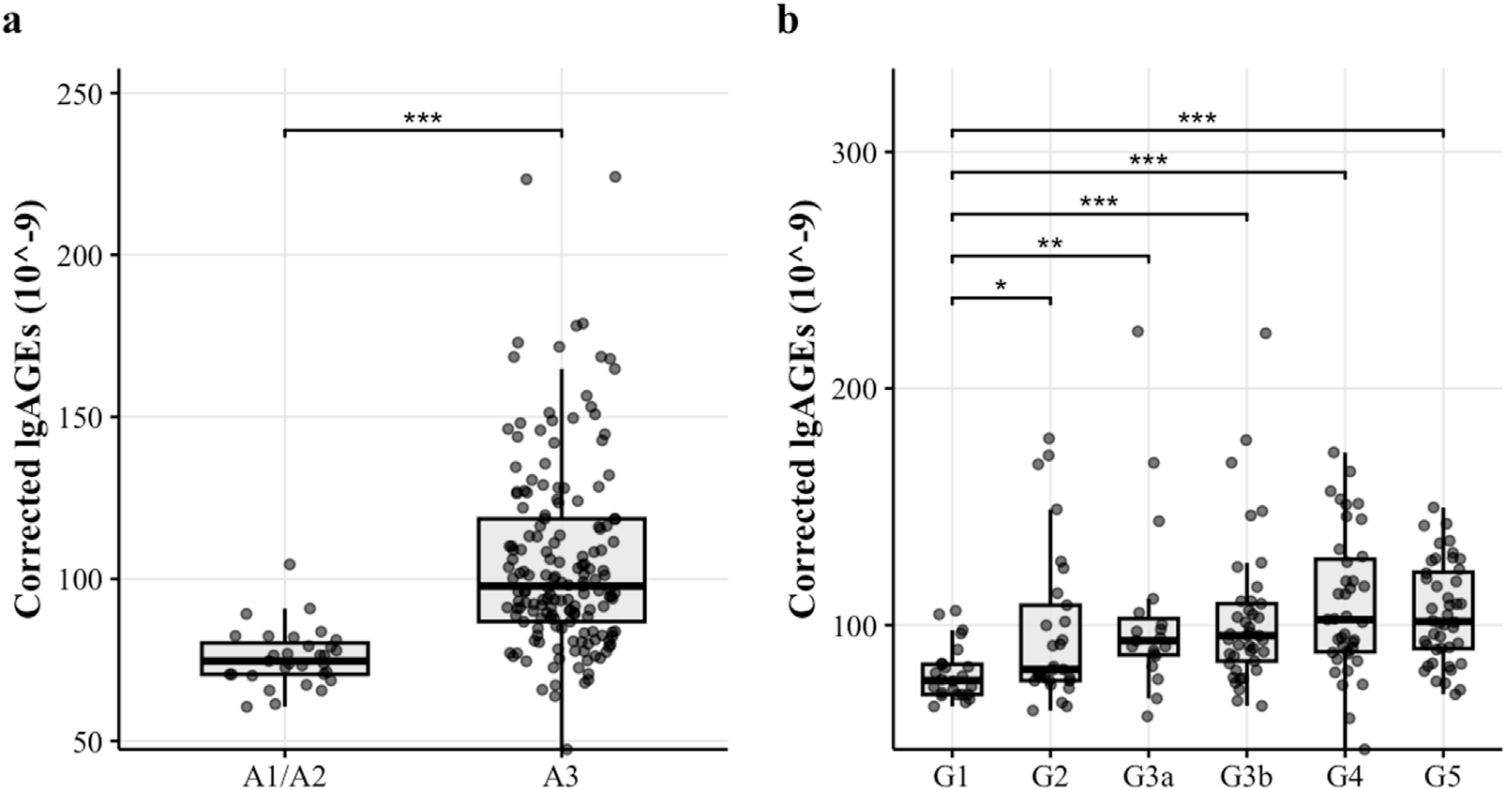
The corrected lgAGEs (10^–9^) content in different stages. Figure notes: **(A)** The corrected lgAGEs content across different UACR stages. Corrected lgAGEs in stages A1 and A2 were significantly lower than those in stage A3 (*p* < 0.05). **(B)** The corrected lgAGEs content across different eGFR stages. Corrected lgAGEs in stages G2, G3a, G3b, G4, and G5 were significantly higher than those in stage G1 (*p* < 0.05).

As shown by Table 4, in the same G stage (except for G3, G4, and G5 stages), there was a significant difference in the corrected lgAGEs content of different A stages (*p* < 0.05). The corrected lgAGEs in stage A3 were notably higher than those in stages A1 and A2 in the same G stage (*p* < 0.05). However, in the same A stage, there was no significant difference in corrected lgAGEs between different G stages (*p* > 0.05). Regarding the G-stage analysis, corrected lgAGEs levels were significantly increased from G1 to G2, G3a, G3b, G4, and G5 stages (median lgAGEs: 75.4, 81.8, 93.4, 94.7, 102.0, and 101.0, respectively; *p* values: 0.028, 0.003, <0.001, <0.001, and <0.001, respectively, compared with G1). Similarly, according to the A-stage analysis results, corrected lgAGEs levels were remarkably lower in A1 and A2 stages compared with those in the A3 stage (median lgAGEs: 74.7 vs 97.8; *p* < 0.001). These findings are illustrated in Figure 2.

**TABLE 4:** Comparison of corrected lgAGEs content in different stages.

eGFR stage(n)	Corrected lgAGEs		z	*P* Value
	Albuminuria stage(n)			
	A1 and A2 (31)	A3 (165)		
G1 (24)	71.946 (70.264–80.736)	86.456 (78.686–97.486)*	−2.817	0.004***
G2 (29)	76.468 (74.689–79.319)	100.648 (80.707–132.372)*	−3.101	0.001***
G3a (19)	89.227 (75.308–90.058)	95.956 (87.108–109.573)	−0.894	0.421
G3b (41)	—	95.507 (84.235–109.573)	—	—
G4 (39)	—	102.440 (88.808–109.846)	—	—
G5 (44)	—	101.489 (89.768–123.128)	—	—
H	2.095	6.529		
*P value*	0.351	0.258		

Note: for the same G stage, compared with A1 and A2 stages, *p < 0.05, **p < 0.01, ***p < 0.001. Due to insufficient data for patients with stage A1 (only 7), the analysis of patients with stages A1 and A2 was combined to reduce potential errors.

### 3.4 Survival analysis of different corrected lgAGEs level

**FIGURE 3:**
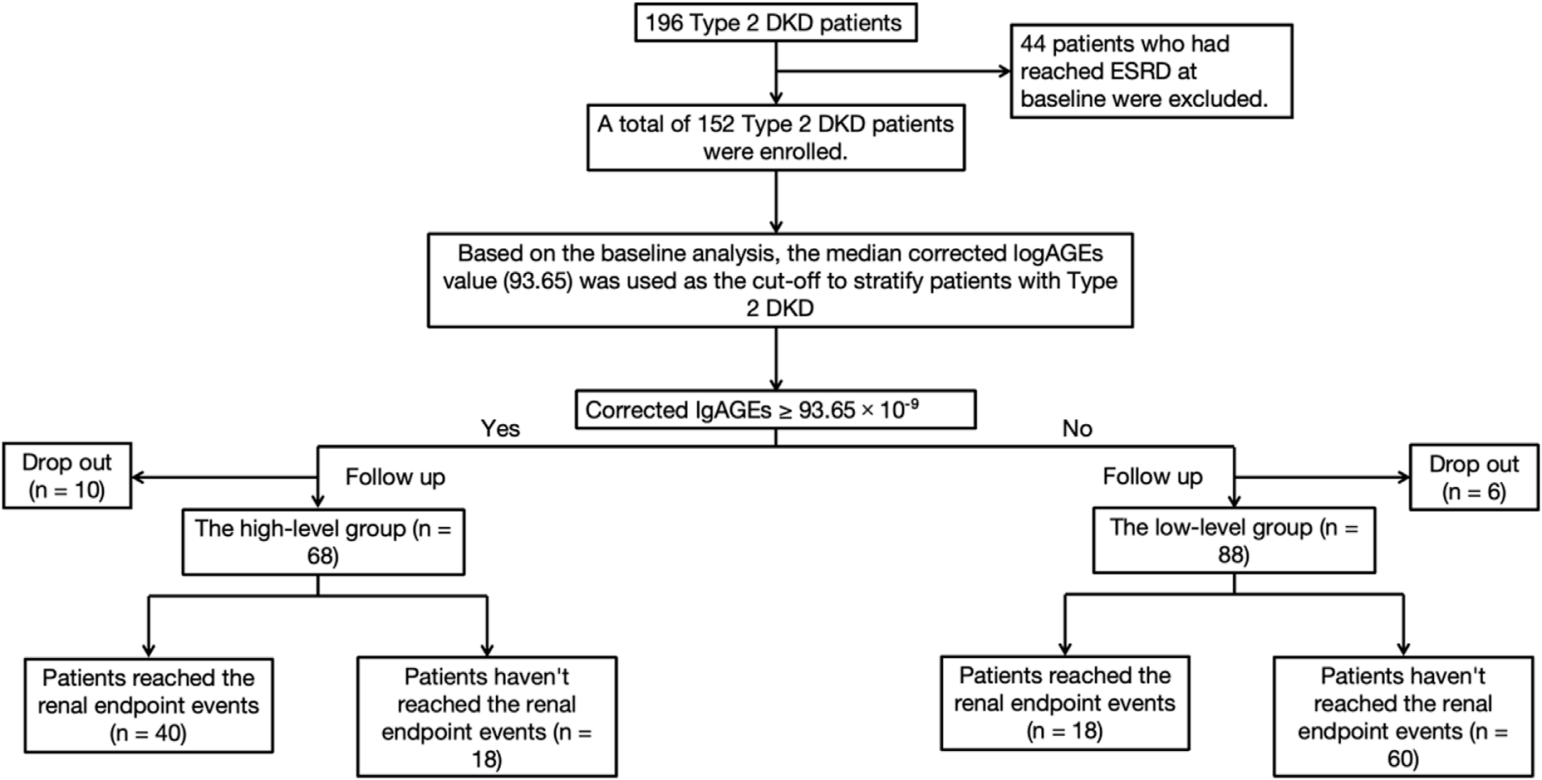
Flow chart of T2DKD patients’ prognosis cohort study.

Patients were allocated into two groups according to their baseline corrected lgAGEs to investigate the influence of AGEs on survival time. Among the 152 participants, the median eGFR was 44.00 mL/min/1.73 m^2^, and the median UACR was 2123.81 mg/g (Supplementary Material). There were 68 patients in the high-level stage group, of which 40 achieved the renal outcome events, defined as doubling of SCr from baseline or entering ESRD, 18 did not, and 10 were lost to follow-up. Among 84 patients in the low-level stage group, 18 attained the renal outcome events, 60 did not, and 6 dropped out. The research flow chart is shown in Figure 3.

**FIGURE 4:**
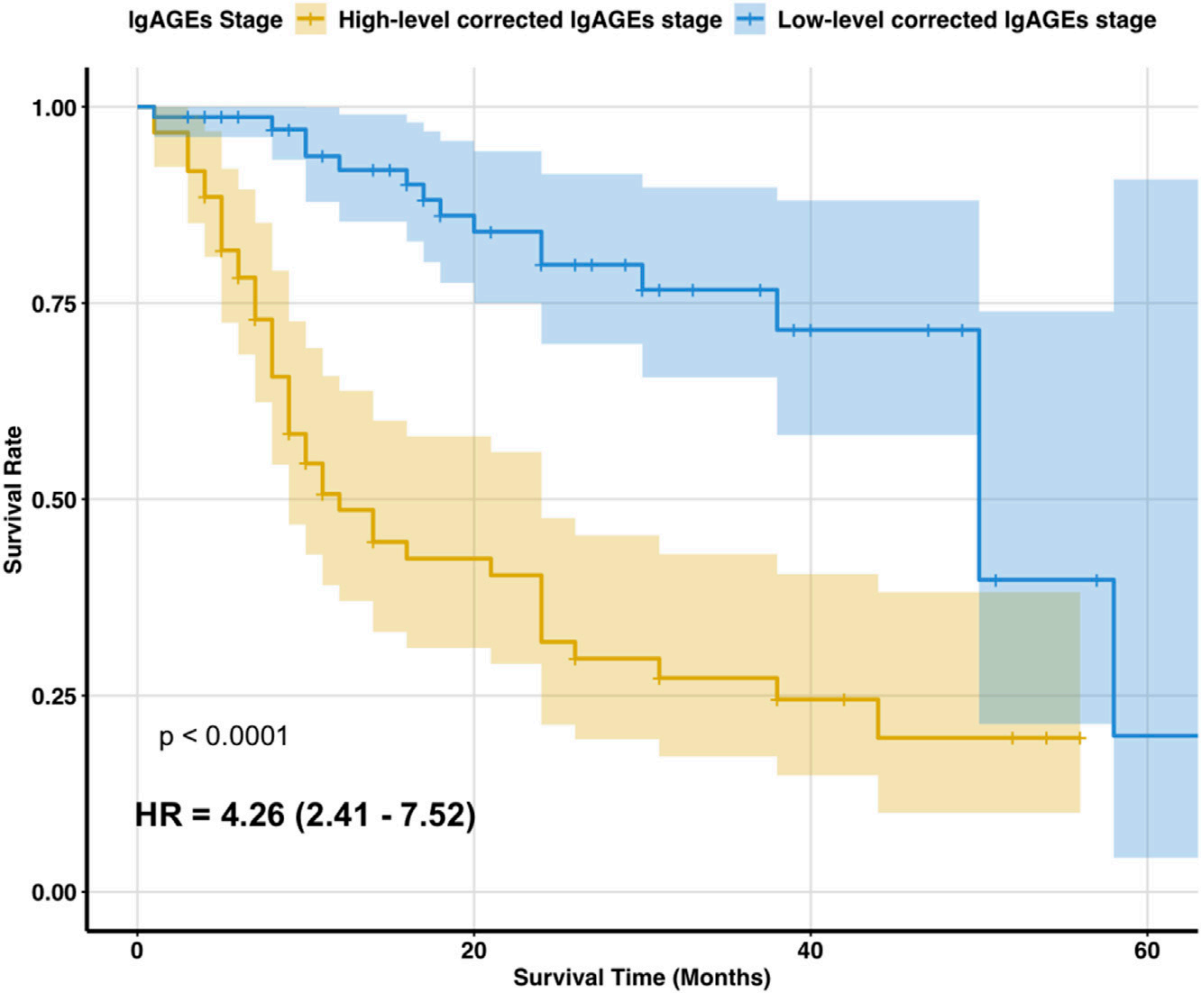
Two stages of survival function curves. Figure notes: Kaplan-Meier survival analysis showed that the median survival time was 12 months for the high-level corrected lgAGEs stage and 50 months for the low-level corrected lgAGEs stage. The survival rate differed significantly between the two stages (*p* < 0.05).

According to the Kaplan-Meier survival analysis results, the median survival time for the high-level stage was 12 months, whereas that for the low-level stage was 50 months, showing a significant difference (*p* < 0.05). The survival function curve is displayed in Figure 4.

### 3.5 Independent renal prognostic factor in patients with T2DKD

Through univariate Cox regression, high-level corrected lgAGEs, HbA1c, SCr, eGFR, UREA, UA, Hgb, UACR, and ACEI/ARB use were identified as significant predictors of renal prognosis. Even after accounting for other factors, high-level corrected lgAGEs [HR: 3.252, 95% CI: 1.461–7.243, p = 0.003] remained a significant predictor of renal endpoint events. Additionally, HbA1c, SCr and ACEI/ARB use were also found to be independent predictors of prognosis (Table 5).

**TABLE 5:** Univariate and multivariate Cox analyses of T2DKD patients’ prognosis.

	Univariate Cox regression		Multivariate Cox regression	
	HR (95% CI)	*p*	HR (95% CI)	*p*
Corrected lgAGEs (High-level vs. Low-level)	3.853 (2.207–6.727)	<0.001	3.252 (1.461–7.243)	0.003
Age (years)	0.993 (0.972–1.014)	0.505	1.017 (0.984–1.052)	0.316
Gender (Male)	1.35 (0.786–2.319)	0.277	1.924 (0.683–5.422)	0.216
Duration of Diabetes	1 (0.997–1.003)	0.967	1.002 (0.998–1.006)	0.42
Drinking	1.41 (0.822–2.419)	0.213	1.372 (0.542–3.473)	0.504
Smoking	1.027 (0.614–1.719)	0.918	1.668 (0.661–4.211)	0.279
eGFR (mL/min/1.73m^2^)	0.962 (0.948–0.976)	<0.001	1.009 (0.975–1.043)	0.622
Glu (mmol/L)	0.899 (0.816–0.991)	0.032	1.114 (0.986–1.259)	0.0841
Hgb(g/L)	0.97 (0.959–0.981)	<0.001	0.993 (0.971–1.016)	0.558
Scr(mmol/L)	1.013 (1.009–1.017)	<0.001	1.011 (1.001–1.022)	0.022
UA (mmol/L)	1.004 (1.001–1.007)	0.003	1.002 (0.998–1.007)	0.318
UACR (mg/g)	1 (1–1)	<0.001	1 (1–1)	0.053
UREA (mmol/L)	1.178 (1.115–1.244)	<0.001	1.089 (0.981–1.21)	0.11
HbA1c (%)	0.673 (0.538–0.841)	<0.001	0.628 (0.482–0.817)	<0.001
24 h-UTP (mg)	1 (1.001–1.002)	<0.001	1 (1–1)	0.495
Hypertension	2.149 (0.777–5.947)	0.141	1.686 (0.387–7.347)	0.487
Dyslipidemia	0.861 (0.503–1.473)	0.585	0.609 (0.285–1.301)	0.22
Coronary Heart Disease	1.111 (0.651–1.899)	0.699	0.872 (0.423–1.798)	0.711
Malnutrition	1.371 (0.815–2.306)	0.234	0.679 (0.338–1.362)	0.276
Stroke	1.005 (0.559–1.806)	0.987	0.658 (0.32–1.353)	0.255
ACEI/ARB	0.412 (0.243–0.697)	<0.001	0.372 (0.187–0.74)	0.005
Non-Insulin Antidiabetic Drugs	1.059 (0.548–2.045)	0.865	0.955 (0.414–2.203)	0.913
Insulin	1.417 (0.829–2.423)	0.202	1.218 (0.549–2.701)	0.628
Antihypertensive Drugs	0.53 (0.316–0.891)	0.01	1.504 (0.756–2.996)	0.245

The HR was derived from Cox regression mode. All factors that may affect the prognosis of DKD patients were included in the Cox regression analysis to screen out the factors that could indicate the prognosis of DKD.

### 3.6 Diagnostic value of corrected lgAGEs in the renal prognosis of T2DKD patients

**FIGURE 5:**
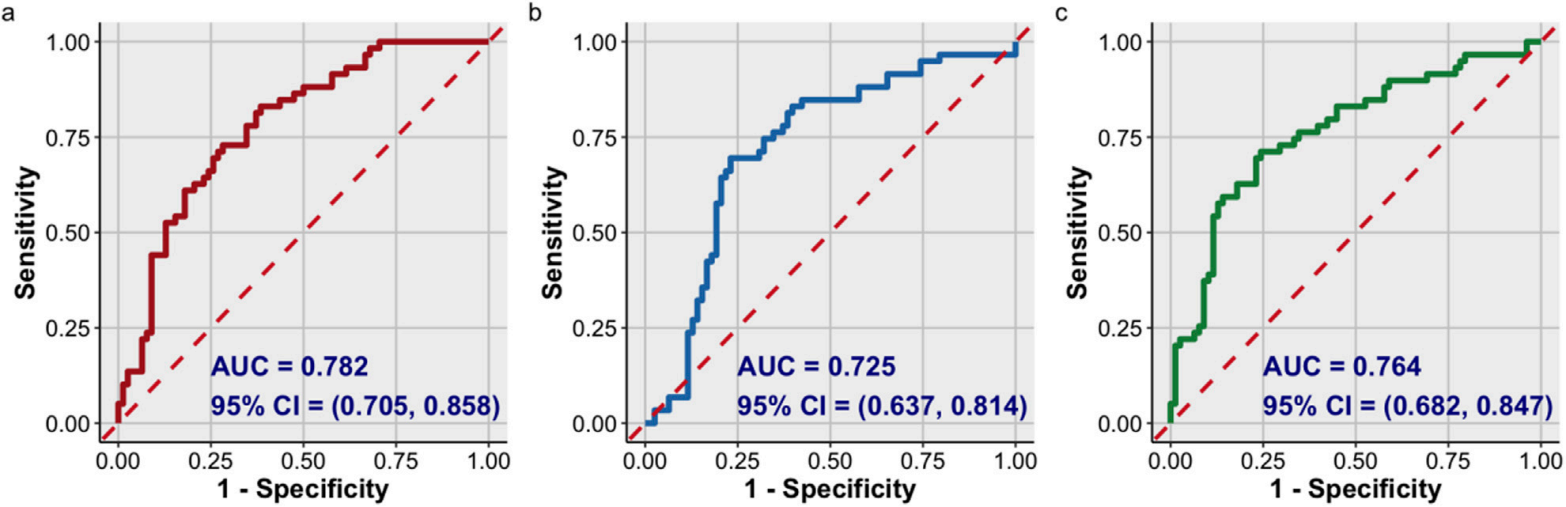
ROC curve analysis of the prognosis of T2DKD. ROC curve of: **(A)** UACR; **(B)** corrected lgAGEs; **(C)** UACR combined with corrected lgAGEs. Data were presented as ROC curves. AUC, area under ROC curve.

As shown in Table 6 and Figure 5, the AUC for the UACR curve was 0.782 (95% CI, 0.705–0.858), with a sensitivity of 82.80% and a specificity of 61.50%. The AUC for corrected lgAGEs was 0.725 (95% CI, 0.637–0.814), with a sensitivity of 69.00% and a specificity of 76.90%. The AUC for UACR combined with corrected lgAGEs was 0.764 (95% CI, 0.682–0.847), with a sensitivity of 70.70% and a specificity of 75.60%. Taken together, UACR combined with corrected lgAGEs exhibited high specificity in predicting prognosis, and UACR had high sensitivity in predicting renal prognosis.

**TABLE 6:** ROC curve analysis of the prognosis of T2DKD.

Factors	AUC	Sensitivity	Specificity	*P* Value	95% CI
UACR	0.782	82.80%	61.50%	<0.001	0.705–0.858
Corrected lgAGEs	0.725	69.00%	76.90%	<0.001	0.637–0.814
UACR + Corrected lgAGEs	0.764	70.70%	75.60%	<0.001	0.682–0.847

Take corrected lgAGEs, as a continuous variable, the ROC, curve showed the results of UACR, corrected lgAGEs, and UACR, combined with corrected lgAGEs’ AUC, sensitivity, and specificity.

## 4 Discussion

AGEs are the end products of a series of complex non-enzymatic glycosylation reactions between the carbonyl group of sugar and the amino group of protein, including carboxymethyl lysine (CML), amylglycoside, aminopyrimidine, pyrroline, methylglyoxal hydroimidazolidone, and other compounds (Shen et al., 2020). Recent studies have highlighted the pivotal roles of AGEs and soluble RAGE (sRAGE) in CKD and ESRD. It has been shown that elevated levels of AGEs are associated with worsened kidney function and increased mortality, particularly in DKD (Steenbeke et al., 2022). AGEs contribute to kidney damage through oxidative stress and inflammation, while sRAGE acts as a protective factor by binding AGEs to mitigate their harmful effects. However, despite the promise of sRAGE as a biomarker, its relationship with kidney function remains complex. Some studies show that it is inversely correlated with kidney function, while others suggest its role as a counter-regulatory response (Dozio et al., 2018). Based on this complexity, AGEs (key components of the AGEs-RAGE axis) have also been demonstrated to hold prognostic value for renal outcomes in DKD patients. Comparatively, the AGEs/sRAGE ratio accounts for the interactive effects between AGEs and RAGE, leading Prasad to propose AGEs/sRAGE as universal biomarkers for the AGEs-RAGE axis-associated diseases (Prasad, 2014). Recent evidence has shown that the AGEs/sRAGE ratio exhibits high sensitivity, specificity, and accuracy in identifying ESRD patients, and its predictive effects are independent of AGE levels (Prasad et al., 2016. However, these findings are confined to cross-sectional studies that distinguish ESRD patients from healthy controls. In contrast, longitudinal evidence has suggested that there is no significant correlation between changes in the AGEs/sRAGE ratio and alterations in eGFR (Molinari et al., 2021). These inconsistent observations underscore the need for further research to elucidate the mechanistic pathways and potential clinical applications of these biomarkers in CKD and ESRD management (Isoyama et al., 2016).

### 4.1 The corrected lgAGEs can better reflect the level of AGEs in the blood

ALB, the most abundant protein in circulation, comprises more than 60% of the total protein in plasma, with its glycosylation level accounting for approximately 80% of all circulating protein glycosylation (Qiu et al., 2021). AGEs crosslink with proteins, resulting in changes in their biophysical properties and impairing their functions (Giglio et al., 2020; Banerjee, 2021). Hypoalbuminemia and ALB-related alterations can affect the concentration of plasma protein glycosylation adducts. Factors that promote ALB metabolism (such as hyperthyroidism, nephrotic syndrome, and glucocorticoid usage) can diminish ALB glycosylation levels (Agalou et al., 2005; Vetter, 2015).

There are several techniques for assessing AGEs in biological fluids and tissues. Among these techniques, immunochemical approaches (such as ELISA and immunohistochemistry) and bioanalytical methods (such as skin autofluorescence, fluorescence spectroscopy, and high-performance liquid chromatography) are most commonly used in clinical practice. However, there is no standardized procedure for clinical AGE detection, nor are there specified units and intervals for the comparison of AGE values between laboratories (Corica et al., 2022). ELISA has been widely used to detect AGEs in diabetes and related vascular complications (Bronowicka-Szydełko et al., 2021; Sabbatinelli et al., 2022; Ding et al., 2024). It's primarily used to analyze protein-bound AGEs.

We hypothesized that ALB influences serum AGE levels. To address this hypothesis, we proposed a novel approach of correcting lgAGEs using ALB, resulting in a new predictor, corrected lgAGEs. This study is the first to validate this approach. According to the results of this study, ALB is an independent determinant of AGEs, supporting the notion that corrected lgAGEs offer a more accurate representation of AGE levels in serum. By accounting for variations in ALB, corrected lgAGEs better reflect the true burden of AGEs, thus providing more reliable markers for clinical assessment.

### 4.2 The corrected lgAGEs are positively correlated with serum creatinine and proteinuria

AGEs, as uremic toxins, are degraded by the receptor after being hydrolyzed by protease or binding with the receptor. The digestive tract and kidney are the primary organs for scavenging AGEs. In healthy adults, approximately one-third of AGEs are eliminated by urine (Twarda-Clapa et al., 2022). In the context of renal injury, the renal filtration and excretion function decreases, leading to reduced excretion of AGEs through the kidney. As a result, there are additional deposits in the tissue, and proteins crosslink, resulting in protein homeostasis imbalance, metabolic imbalance, and interruption of the cellular signal pathway (Agalou et al., 2005). However, our study discovered that AGEs were positively associated with eGFR and negatively associated with serum creatinine, which is consistent with previous clinical research but appears to contradict intuitive suppositions (Bronowicka-Szydełko et al., 2021; Koska et al., 2022). The association of AGEs with kidney disease extends beyond directed causation. Nonetheless, it is believed to be associated with the rapid synthesis of plasma proteins. AGEs may be a protective mechanism that permits long-lived proteins (such as extracellular matrix proteins) to withstand glycan-induced oxidative damage (Rungratanawanich et al., 2021).

After stratifying all DKD patients into G and A stages, it was found that the content of corrected lgAGEs showed different trends at different stages of DKD. In the same G stage, corrected lgAGEs showed a rising trend as the A stage increased, indicating that the content of corrected lgAGEs was positively correlated with the degree of proteinuria. Additionally, in the same A stage, corrected lgAGEs tended to increase with a decrease in eGFR. As indicated by the correlation analysis results, corrected lgAGEs were positively linked with SCr and UACR, which appears to contradict previous conclusions. This may be owing to the realization that UACR has an independent impact on corrected lgAGEs. As has been evidenced previously, dialysis patients with proteinuria have lower plasma amyl glycoside levels than non-proteinuria patients, indicating that proteinuria affects serum AGE levels (Galli, 2007). In an experiment on streptozotocin (STZ) rats, the increase of CML excretion in urine is related to the increase of urinary microalbumin, and the increase of urinary CML occurs before proteinuria changes (Coughlan and Forbes, 2011). This further supported our findings that UACR had a greater impact on corrected lgAGEs than SCr.

### 4.3 The corrected lgAGEs might be potential biomarkers for DKD prognosis

Several factors influence the prognosis of DKD. Among these, UACR and eGFR are the most critical biomarkers for assessing DKD severity and prognosis. However, relying solely on these two indicators is insufficient to accurately predict DKD progression (Dong et al., 2022). Although measurements of UACR and eGFR provide a snapshot of current renal health, they are limited by the inherent variability in GFR and albuminuria levels, which are prone to minor fluctuations that may not reflect long-term GFR trajectories or changes in albuminuria (Yamanouchi et al., 2024). Furthermore, the heterogeneity of DKD progression highlights significant inter- and intra-individual variability in GFR decline and albuminuria levels, which further limit the predictive value of these biomarkers.

In contrast, AGEs are intricately involved in the pathogenesis of DKD through their complex causal relationships with proteinuria and renal function decline. AGEs can cause podocyte injury, contributing to proteinuria (Nishad et al., 2021; Vlassara et al., 1994). Meanwhile, it's also influenced by impaired renal clearance, resulting in their accumulation (Steenbeke et al., 2022). This accumulation creates a vicious cycle, exacerbating DKD progression. Incorporating AGEs into existing risk models for DKD enhances the ability to predict sustained renal function loss (Koska et al., 2022). Notably, the predictive utility of AGEs extends to individuals with normal renal function (eGFR ≥90 mL/min/1.73 m^2^) and normal albuminuria, highlighting their potential as biomarkers for adverse renal outcomes in early stages of kidney disease (Prasad, 2014; Saulnier et al., 2016).

We consider that serum AGEs are related to DKD progression. In recent years, with the rising interest in “metabolic memory”, a growing number of studies have revealed that AGEs may be associated with the development of renal function in diabetes patients (Jin et al., 2022a). In DKD, the production of AGEs increases, but their breakdown and excretion decrease; additionally, they continue to accumulate in tissues and circulation, contributing to ongoing kidney damage. Svetlana Baskal et al. have found that lower urinary excretion of AGEs is associated with higher all-cause mortality in adult kidney transplant recipients (Baskal et al., 2021). Moreover, in a previous study on American Indians with type 2 diabetes, blood levels of serum carboxyethyl lysine and methyl ethyl two-hydroxy imidazolone (which belong to AGE components) are linked to an increased risk of kidney function loss (Saulnier et al., 2016). After accounting for the risk variables that influence the prognosis of kidney disease, CML hydroimidazolone greatly improves prediction (Nishad et al., 2021). As evidenced by previous cross-sectional studies, circulating AGEs in patients with type 1 DM decrease with enhanced glycemic control. Even after adjusting for glycemic control markers (such as HbA1c), AGEs are still strongly associated with the progression of microvascular complications in type 1 DM (Monnier et al., 2022). These findings supported our results. Briefly, this study discovered that high-level corrected lgAGEs were independent risk factors affecting the prognosis of DKD; UACR combined with corrected lgAGEs for DKD prediction had a high specificity (up to 75.60%), implying that high-level corrected lgAGEs at baseline may indicate a poor prognosis of DKD.

Consistent with the importance of AGEs in DKD prediction, animal studies have demonstrated that boosting the breakdown or excretion of AGEs, as well as blocking the AGEs-RAGE axis, can help delay DKD progression (Azegami et al., 2021; Li et al., 2021). As drugs targeting AGEs have not yet been widely used in clinical trials, it is unclear whether they can prevent the decline of renal function among individuals by inhibiting AGEs (Tang and Kalim, 2022).

In our investigation, AGEs had no predictive value for renal endpoint events in DKD (data not shown). However, corrected lgAGEs had predictive value. After controlling for HbAlc, SCr, and UACR, multivariate Cox analysis revealed that high levels of corrected lgAGEs continued to have a predictive effect on renal endpoint events in T2DKD. As demonstrated by receiver operating characteristic (ROC) curves, UACR combined with corrected lgAGEs had high prognostic specificity, whereas UACR was extremely prognostic-sensitive. This may be because most previous studies focused on patients with early DM who had high eGFR and ALB, whereas our study sample included both hypoproteinemic and non-hypoproteinemic individuals. This indicated that when measuring AGEs, we should consider the impact of ALB. Although corrected lgAGEs do not surpass UACR in prognostic value for renal outcomes, they exhibit greater specificity. Over time, non-albuminuric CKD has emerged as the predominant DKD phenotype among type 2 diabetes patients with eGFR decline (Jia et al., 2022). Studies have indicated that individuals with reduced GFR but no albuminuria face a higher risk of CKD progression (Jin et al., 2022b). This suggests that relying solely on UACR to evaluate the risk of DKD patients with normal albuminuria may lead to the omission of critical prognostic information. Moreover, due to individual differences and the influence of multiple pharmacological agents, UACR is subject to significant variability, resulting in fluctuations in clinical trajectories (MacIsaac et al., 2014; Jongs et al., 2021; Ceccarelli et al., 2022). In contrast, AGE levels remain stable over time. For patients with normoalbuminuric DKD, monitoring AGEs may provide additional prognostic benefits by capturing risks that may be overlooked by UACR alone.

It is now widely recognized that improving glycemic management can help control the progression of diabetes complications (Mottl et al., 2022). Other studies have demonstrated that glycemic management has long-term consequences; strict glycemic control in the short term cannot prevent or delay the onset of ESRD in individuals with DKD (Takao et al., 2019; Kornelius et al., 2020). Unlike previous studies, our findings showed that HbA1c was a protective predictive factor for DKD. This may be because our research samples are primarily elderly DKD patients.

Our research has several advantages. First, natural log-transformed AGEs were employed in this study and adjusted with ALB. This study was the first DKD clinical study to closely connect blood AGEs to serum ALB. Second, the stage of DKD in the included patients was not restricted, and our research sample comprised patients with moderate to severe DKD who had significant proteinuria and hypoproteinemia. The average eGFR for the study population was 35.50 mL/min/1.73 m^2^, with a UACR of 2802.41 mg/g and an ALB of 32.90 g/L.

However, our research has the following limitations. First, ELISA was used to detect serum AGEs, which also demonstrated the novelty of this study in choosing the ALB correction method. However, there are insufficiencies in the calculation of statistical quantities. Second, as this was a monocentric, small-sample clinical study, circulating AGEs were detected in only 196 patients at baseline. There was no monitoring of AGE excretion in the urine, no quantitative drug use, and no adequate nutritional indicators (such as Fat-Free Mass Index). Therefore, it was impossible to determine the potential impact of drug use or nutritional conditions on AGEs. Third, the high-level and low-level corrected lgAGEs groups are not fully matched between the eGFR and the UACR, making it more prone to classification errors. Fourth, corrected lgAGEs in our results have a higher specificity. Although their AUC is not higher than that of UACR, they provide a better direction for investigating the prospective performance of corrected lgACEs in DKD patients with normal protein proteinuria. This could help reduce the impact of UACR in future studies.

## 5 Conclusion

This prospective cohort study demonstrated that serum AGEs, when adjusted for serum albumin levels, yield albumin-corrected lgAGEs as a predictive marker for the prognosis of type 2 DKD. The combination of albumin-corrected lgAGEs with other markers further improved the early identification of high-risk individuals with DKD. Nevertheless, the small sample size and single-center design may limit the generalizability of these findings. While these results underscore the potential utility of AGEs in DKD management, larger, multicenter studies are needed to validate the robustness and clinical applicability of albumin-corrected lgAGEs as a biomarker. Future research should also investigate the longitudinal predictive value of AGEs and their integration with other biomarkers to enhance risk stratification and guide disease management in DKD.

## Data Availability

The raw data supporting the conclusions of this article will be made available by the authors, without undue reservation.
