# Supplementary material for: Serum advanced glycation end products as a putative biomarker in Type2 DKD patients’ prognosis
Source: Front Physiol. 2025 Jan 31;16:1541198. doi: 10.3389/fphys.2025.1541198 (PMC11825471; doi:10.3389/fphys.2025.1541198)
Supplement: Supplementary file 1 [file Table1.docx]

**Supplementary Materials**

Table1 Multiple linear regression analysis of AGEs

| Independent variable | Non-standardized β | Standard error | Beta | t | *P* value |
| --- | --- | --- | --- | --- | --- |
| Smoking | -199.479 | 116.551 | -0.119 | -1.712 | 0.089 |
| SCr(umol/L) | -0.817 | 0.419 | -0.195 | -1.952 | 0.052 |
| eGFR(mL/min/1.73m^2^) | -1.512 | 3.17 | -0.059 | -0.477 | 0.634 |
| UA(mmol/L) | -0.745 | 0.714 | -0.079 | -1.044 | 0.298 |
| UREA(mmol/L) | -0.544 | 0.435 | -0.087 | -1.252 | 0.212 |
| Hgb(g/L) | 1.634 | 3.13 | 0.048 | 0.522 | 0.602 |
| Alb(g/L) | 16.647 | 8.262 | 0.16 | 2.015 | **0.045*** |

Table2 Survival analysis characteristics of the 152 T2DKD patients

| Variables | All(n=152) | low-level corrected lgAGEs stage(n=84) | high-level corrected lgAGEs stage(n=68) | t/ Z/χ2 | *P* value |
| --- | --- | --- | --- | --- | --- |
| Age(years) | 60.50(53.00-70.00) | 62.50(53.00-71.00) | 60.00(50.00-65.75) | -1.465b | 0.143 |
| Gender (Male,%) | 110(72.40%) | 63(75.00%) | 47(69.1%) | 0.650c | 0.420 |
| Diabetes course(months) | 200.00(132.00-251.25) | 204.00(144.00-264.00) | 192.00(120.00-240.00) | -0.173b | 0.083 |
| Smoking (Yes ,%) | 65(42.80%) | 36(42.90%) | 29(42.6%) | 0.001c | 0.979 |
| Drinking (Yes ,%) | 45(29.60%) | 21(25.00%) | 24(35.30%) | 1.911c | 0.167 |
| Diabetic retinopathy (Yes ,%) | 100(65.8%) | 49(61.30%） | 51(78.50%） | 4.964c | 0.026* |
| Diabetic peripheral neuropathy (Yes ,%) | 73(48.00%) | 41(53.20%) | 32(49.20%） | 0.228c | 0.633 |
| Diabetic foot (Yes ,%) | 9(5.90%) | 4(5.20%） | 5(7.8%） | 0.401c | 0.527 |
| Hypertension (Yes ,%) | 135(88.81%) | 61(91.00%) | 74(88.10%) | 0.342c | 0.559 |
| Dyslipidemia(Yes ,%) | 99 (65.13%) | 52 (61.9%) | 47 (69.12%) | 0.573c | 0.449 |
| Coronary Heart Disease(Yes ,%) | 49 (32.24%) | 27 (32.14%) | 22 (32.35%) | ＜0.001c | 0.996 |
| Malnutrition(Yes ,%) | 65 (42.76%) | 33 (39.29%) | 32 (47.06%) | 0.637c | 0.425 |
| Stroke(Yes ,%) | 37 (24.34%) | 20 (23.81%) | 17 (25%) | 0.17c | 0.681 |
| AGEs (pg/ml) | 1458.519(1195.846-1740-525) | 1458.519(1072.773-1795.683) | 1452.655(1242.483-1663.139) | 0.239b | 0.811 |
| Corrected lgAGEs(10^-12^) | 91.420(78.529-109.846) | 79.841(73.825-88.245) | 113.168(101.267-146.178) | 10.583b | ＜0.001*** |
| HbA1c(%) | 7.000(6.100-8.000) | 7.20(6.30-8.10) | 6.800(5.800-7.850) | -2.045b | 0.041* |
| Glu(mmol/L) | 7.400(6.090-9.580) | 7.645(6.360-10.290) | 7.290(5.580-8.210) | -2.208b | 0.027* |
| SCr(umol/L) | 141.100(89.000-195.700) | 108.350(77.950-178.800) | 160.900(114.775-238.200) | 3.665b | ＜0.001*** |
| eGFR(mL/min/1.73m^2^) | 44.000(29.050-76.550) | 56.700(31.950-89.650) | 38.750(23.375-54.150 | -3.555b | ＜0.001*** |
| UA(mmol/L) | 386.350(340.275-457.325 | 389.550(340.275-457.325) | 385.350(337.325-459.650) | 0.080b | 0.937 |
| UREA(mmol/L) | 9.200(7.135-12.845) | 8.510(6.037-12.270) | 9.835(8.082-13.407) | 2.499b | 0.012* |
| CHO(mmol/L) | 4.860(3.835-6.142) | 4.155(3.487-5.017) | 5.870(4.797-7.055) | 5.461b | ＜0.001*** |
| TG(mmol/L) | 1.755(1.147-2.582) | 1.590(1.142-2.477) | 1.840(1.142-2.735) | 0.734b | 0.463 |
| UACR(mg/g) | 2123.810(607.617-4370.827) | 836.175(150.014-2082.593) | 4498.852(3171.350-6513.798) | 8.441b | ＜0.001*** |
| 24h-UTP(mg) | 2275.450(816.100-5694.600) | 1320.800(282.450-2298.075) | 5581.000(2997.300-8677.500) | 7.513b | ＜0.001*** |
| ALB(g/L) | 33.858±8.275 | 39.639±4.800 | 26.716±5.667 | 14.956a | ＜0.001*** |
| Hgb(g/L) | 119.070±22.667 | 125.070±21.674 | 111.650±21.79 | 3.788a | ＜0.001*** |
| ACEI/ARB (Yes ,%) | 84 (55.26%) | 53 (63.1%) | 31 (45.59%) | 3.978c | 0.046* |
| Non-Insulin Antidiabetic Drugs (Yes ,%) | 129 (84.87%) | 70 (83.33%) | 59 (86.76%) | 0.129c | 0.719 |
| Insulin (Yes ,%) | 97 (63.82%) | 52 (61.9%) | 45 (66.18%) | 0.141c | 0.708 |
| Antihypertensive Drugs (Yes ,%) | 86 (56.58%) | 51 (60.71%) | 35 (51.47%) | 0.958c | 0.328 |
| SGLT2i((Yes ,%) | 28 (18.42%) | 20 (23.8%) | 8 (11.76%) | 0.989c | 0.32 |

*Data are presented as the mean ± standard deviation or median (IQR: Q1–Q3) for continuous variables and percentage for categorical variables.*

*a Student's t-test analysis,*

*b Mann-Whitney U analysis,*

*c chi-square test analysis.*
